# Supplementary material for: The Formyl Peptid Receptor Ligand Ac2-26 Improves the Integrity of the Blood−Brain Barrier in the Course of Pneumococcal Meningitis
Source: Cells. 2024 Dec 19;13(24):2104. doi: 10.3390/cells13242104 (PMC11674053; doi:10.3390/cells13242104)
Supplement: Supplementary file 1 [file cells-13-02104-s001.zip › cells-3280620-supplementary.pdf]

Supplemental Table S1. Summary of the different results for the time courses of the BBB markers

	Fibrinogen<br>Positive Area [%]	PECAM-1<br>Mean grey value<br>in calibrated units	Claudin-5<br>Mean grey value<br>in calibrated units
WT control	0.034 ± 0.024	1.598 ± 0.788	54.49 ± 2.936
WT 30 h infected	0.451 ± 0.137	10.54 ± 2.568	30.10 ± 1.545
WT 48 h infected	1.119 ± 0.321	**19.39 ± 3.314*** **	**20.22 ± 3.019** **
WT mortality	**9.031 ± 3.53** **	5.878 ± 1.828	**16.73 ± 1.893*** **

Data are presented as mean with standard error of the mean. The significant differences compared to the uninfected control are highlighted (n≥4 each group; One-way ANOVA followed by Bonferroni test; ** - p < 0.01; *** - p < 0.001).

Supplemental Table S2. Summary of the different results for the influence of FPRs and its ligand A2-26 for the BBB markers

	Fibrinogen<br>Positive Area [%]	PECAM-1<br>Mean grey value<br>in calibrated units	Claudin-5<br>Mean grey value<br>in calibrated units
WT control	0.06 ± 0.018	1.598 ± 0.788	54.49 ± 2.936
WT control + Ac2-26	0.082 ± 0.025	0.302 ± 0.214	**39.060 ± 5.057** **
WT infected	**0.451 ± 0.137*** **	7.915 ± 4.485	**30.10 ± 1.545**** **
WT infected + Ac2-26	0.253 ± 0.106	1.877 ± 0.886	**38.51 ± 3.264*** **
FPR1-KO control	0.019 ± 0.006	**11.473 ± 1.981#**	**35.604 ± 3.969#**
FPR1-KO control + Ac-26	0.015 ± 0.006	**24.493 ± 3.675** **	**24.943 ± 5.385§**
FPR1-KO infected	**0.285 ± 0.104***	5.099 ± 1.584	32.25 ± 1.943

FPR1-KO infected + Ac2-26	0.022 ± 0.009	**18.345 ± 4.949+**	42.11 ± 1.758
FPR2-KO control	0.029 ± 0.015	6.758 ± 2.717	**57.65 ± 6.865§**
FPR2-KO control + Ac-26	0.055 ± 0.025	1.615 ± 1.242	**37.229 ± 2.728****
FPR2-KO infected	0.228 ± 0.120	0.48 ± 0.351	**27.87 ± 1.092******
FPR2-KO infected + Ac2-26	0.210 ± 0.074	4.206 ± 2.349	**28.33 ± 1.771******

Data are presented as mean with standard error of the mean. The significant differences compared to the respective control (* - $p < 0.05$; ** - $p < 0.01$; *** - $p < 0.001$; **** - $p < 0.0001$), to the WT control ($^{\#}$ - $p < 0.05$), to the FPR1-KO control or FPR1-KO control + Ac2-26 (§ - $p < 0.05$) or to the FPR1-KO 30 h infected (+ - $p < 0.05$) are highlighted (n≥4 each group; One-way ANOVA followed by Bonferroni test).
